# Two Cases of Hydrophobia

**Published:** 1812-11

**Authors:** John Tytler

**Affiliations:** London


*To the Editors of the Medical and Physical Journal.*

GENTLEMEN,

HAVING received the following Cases from my brother, Dr. Robert Tytler, of Calcutta, I take the liberty of transmitting them to you for insertion in your Journal, should you think them of sufficient importance. I shall make no further remark on them than to observe, that they have been made public in India, and are admitted to be strictly correct in the relation.

I am, Gentlemen,

*London,* Your's, &c.

*Sept.* 20, 1812. JOHN TYTLER.

*Fatal*

### *Fatal Case of Hydrophobia, by* Mr. Tymon; *with Remarks, by* Dr. A. Berry.

### THE UNSUCCESSFUL CASE.

John Irwin, private dragoon, on the 6th of October, in the morning, when I first saw him, was convulsed all over his body, the muscles of his neck unusually agitated, pulse at times quick, and afterwards ceasing altogether for a short lapse; ordered him into the hospital. On being brought there, found no alteration in his symptoms, directed an anti-spasmodic draught, which I offered in person: this he first viewed in silence; but, when pressed to take it, and on discovering it to be a liquid, his convulsions increased with redoubled violence, sweats broke out profusely, attended with difficult respiration. He said the light caused him great uneasiness, consequently the light was excluded. He was observed to froth at the mouth, and his insuperable aversion to any thing in the form of fluid, awoke in my mind the belief that all these direful symptoms must be connected with, or in fact be the result of, canine madness. Unhappily, on inquiry, this supposition was not groundless, for the patient acknowledged his being bit on the 15th July last by a dog in the barracks; his raving rather increased, but his wife, an European, corroborated the fact. I did my utmost to induce him to swallow the draughts alluded to, but without success. He was sensible to reasoning at times, and abused dogs, lamenting his situation; but nothing liquid would prove acceptable: whenever liquids were offered, he took them into his hands, attempting putting the fluids to his mouth, and immediately dashed them on the ground, and then his spasms commenced. He allowed me to bleed him; I took about 15 oz. of blood, and bound up his arm; this produced no alteration in his aversion to fluids; ordered an injection of 300 drops of laudanum, and to rub in a drachm twice every hour. He expired five hours after being admitted.

On opening the body, the appearances were remarkable. The stomach was inflamed; several blood vessels were ruptured, and the stomach loaded with bile; the intestines appeared to have undergone no change; the liver was slightly altered from its natural color; the gall-bladder enlarged considerably, and full of bile very hot to the touch; the lungs were not changed in any manner, nor could I discover any thing preternatural in the thorax; his eyes were swelled; and the blood vessels on the tunica conjunctive enlarged beyond conception. On opening the head, nothing appeared which could induce a belief that any part of the brain or its vessels had suffered; the tongue was swelled, and so were the fauces, and inflamed.

SUCCESSFUL

SUCCESSFUL CASE OF HYDROPHOBIA.

Benjamin Mason, a farrier, aged 34, was seized with violent spasms on the 7th October, when I saw him. I found him violently agitated, and screaming loudly; eight of the strongest men in the regiment were required to keep him on his cot. He clinched his fist at times, and made efforts to seize every thing he saw. In the midst of the paroxysm, he said carriages, horses, animals of every description, were floating before him in the air. He was covered all over with sweat, his eyes at times staring, and at other times melancholy. He gnashed his teeth in a manner not to be described; his neck was swelled, pulse very fast, light distressing, pain in his head and temples increasing; he called for drink, but, the instant he heard them pouring water into a tumbler, his wildness increased; he then beckoned for it, when it came nigh him he shook his hands, trembled, and shivered. I made inquiries if he had been bitten by a dog at any period; his comrades acknowledged he was. One hour after I saw him, his ravings and efforts to disengage himself from his keepers became so violent, as to render it necessary to tie him to his cot; accordingly he was secured by several coils of bed-tape. I discovered he was bit on his left thumb, therefore no obscurity now remained as to the nature of his complaint. I began by bleeding him until *scarcely a pulsation was to be felt in either arm.* During the operation, he made several efforts to bite me; but the quantity of blood taken away naturally reduced his efforts. I now renewed offering the draught, which consisted of 100 drops of laudanum in mint water; he endeavoured to reject it, but I separated his jaws by means of a small piece of wood introduced between them, and poured the draught into his mouth, which he swallowed by keeping his head in a recumbent posture, notwithstanding the efforts he made to reject it. His condition was so much debilitated from loss of blood, as to enable me to effect it.

In the mean time, ordered injections of 300 drops of laudanum every second hour, and a drachm of mercurial ointment to be rubbed in every fourth hour.

P.M. four o'clock. Is now in a slumber; at half past five he awoke with slight efforts to separate himself from his bindings; pain in his head excruciating; shaved his head, and blistered it all over; mercurial frictions to be continued; still has an aversion to fluids, used the same means as before; draught and glysters to be repeated.

P.M. nine o'clock. Slept for two hours, and appears to be refreshed; complains of a lassitude and sickness at stomach; mach; speaks rationally; offered him congee water, which he kept in his mouth for a short time, swallowed a little, and discharged the remainder; pulse rising; repeat the frictions; to take a pill of four grains of calomel and two grains of James's powder three times, and repeat the injection twice during the night.

8th. Pain in his head subsided; extremely debilitated, but rational; calls for drink, which, with some hesitation, he puts to his mouth, and swallows with a slight noise in his throat; pulse low; mercurial frictions, as before described, to be continued; a pill of calomel four grains, and of opium and James's powder two grains, every second hour; opiate injections to be repeated.

P.M. Pain in his forehead become excruciating, was tranquil otherwise during the day; pulse 79; a blister to be applied to his forehead; to repeat the pills three times in the night, and to rub in a drachm of mercurial ointment every second hour.

9th. Very easy; relieved from pain and uneasiness in his head; had only one evacuation for the last twenty-four hours; castor oil, one ounce, to be taken immediately; frictions to be continued.

P.M. Gums getting tender; feels no horror at the sight or approach of liquids; pulse rather hurried; to rub in a drachm of the ointment thrice during the night; pills to be continued; bathed his feet in warm water.

10th. Fugitive dislike to fluids; when pressed, swallows congee water, and took one glass of wine; pulse rising and regular; gums tender; continue frictions; repeat pills.

P.M. Bathed his feet as before.

11th. No mercurials in any shape; complains of weakness; his countenance is fallen, but appearances of doing well are manifest; spits a great deal; continue pills and frictions.

P.M. No alteration; repeat pills twice during the night.

12th. Very easy; gums inflamed.

13th. Very easy; 14th, ditto; 15th, ditto; 16th, ditto; 17th, ditto; 18th, ditto; 19th, ditto; 20th, discharged from the hospital in a perfect state of convalescence, and he has since resumed his duties (Nov. 1st).

(Signed) F. TYMON,
Assistant Surgeon 22d Dragoons.

*Observations by* A. Berry, M.D.

That this was a real cure of hydrophobia cannot be doubted. To satisfy me the more fully on this subject, a statement has been forwarded from Captain Broome, commanding manding the detachment, depicting the situation of Farrier Mason, as stated by Mr. Tymon. Captain Broome says that Mason, after the bleeding, became somewhat composed, and held out his hand, saying "I was bit here sir," and Captain Broome states "I plainly saw a *dark spot*, as of a wound, on the inside of his finger."

A certificate, from Henry Jackson, serjeant, specified that he and Irwin were bit on the same day, in the beginning of August, and Mason a day or two after.

As to the treatment, it must be immediately remarked by the medical reader of these two cases, that the bleeding in John Irwin's case, carried only to the extent of fifteen ounces, did not arrest in any degree the rapid progress of the disease; and that the bleeding of Benjamin Mason, continued *until scarcely a pulsation in either arm was to be felt*, saved his life by diminishing violent action, and admitted the effect of medicines that in all former experience had uniformly failed.

It also appears from these two cases, and several unsuccessful ones I have seen, that the hydrophobia from the bite of a rabid animal is of short duration, all those that I have seen having died within twenty-four or thirty-six hours at farthest from the appearance of the disease; and Benjamin Mason was evidently nearly cured the evening of the day he was attacked, for, at nine o'clock at night of the 7th Oct, he could keep congee water in his mouth, and swallowed a little.

It would also appear that this disease may sometimes put on so mild a form as not to be dangerous, as appears by two other cases from Mr. Tymon; one of Corporal Rice, who was bit severely in the hand by a dog about the middle of May, and another of Sergeant Jackson, who was bit on the same day that John Irwin was, about the beginning of August, instead of the 15th of July, as stated in Irwin's case.

Corporal Rice was admitted into the hospital on the 6th of Oct. in the evening, for symptoms indicating hydrophobia, appearing agitated and dejected, complaining of excruciating pain of his head, refusing to drink water when offered to him, pushing it from him at the same time in a gentle manner, accompanied with a deep sigh. He walked about the room, and his ideas were somewhat deranged; refused to drink water for the whole of the 6th, or even to suck limes, though thirsty. His head was blistered, and mercury used both internally and externally by friction. The hydrophobic symptoms in this case ceased in the evening of the succeeding day.

Serjeant Jackson, bit by the same dog that bit Benjamin Mason,

Mason, was admitted into the hospital on the evening of the 7th of Oct. He was one of the number that secured Mason to his cot; he then ridiculed the idea of any danger to himself. When admitted, he complained of tormenting pain in his head, impatience of light, started and was agitated by any sudden gust of wind blowing on him, had a great flow of saliva, was much distressed and agitated at even the sight of water poured out, pulse regular, partial sweats, drowsy and melancholy, eyes almost fixed and very much inflamed. By blisters to his head, and mercury internally, and by friction and purgative injections he was relieved, and in the evening of the succeeding day he called for food, and drank congee water without difficulty.

These few detailed facts tending to confirm the short period of the disease, I consider of value, as giving confidence to the practitioner, and hope to the patient. By showing when the progress of the disease can be arrested, a cure may be effected.

The state of John Irwin's stomach, if solely caused by hydrophobia, would indicate the inflammatory nature of the disease, and its principal seat of action from the fauces to the stomach, rendering it probable that all irritating medicines are improper, and likely to be hurtful, until that inflammatory action is diminished. I mention this particularly, from verdigrease in considerable quantity having been stated in an American paper to have cured this disease; but I have seen no cases to satisfy me of the utility of this medicine, or that it was ever given with effect in any severe case of hydrophobia.

I will make a few extracts from authors to shew how remarkable the successful treatment of hydrophobia must be considered. The celebrated Dr. Fothergill published a case of hydrophobia from the bite of a cat, in 1774, which terminated fatally in about forty-eight hours; and, in his notes on the case, when speaking of a cure for the canine madness, he says, "the reader will find, perhaps, nothing more of certainty in this respect, than that *all the remedies*, proposed either as preventatives or cures, are found by experience to be altogether *ineffectual.*"

This patient was blooded, but only six ounces of blood at first are stated to have been abstracted. He was a second time bled, as Dr. Fothergill states, standing, as his strength would bear; and, in the evening, the patient received his physicians with the utmost transport of joy, describing, in strong terms, the pleasure and benefit he received from the warm bath, and the hopes he now conceived of a speedy recovery. The bleeding would, in this case, appear to have been too long delayed, for he died exhausted in about seven or eight hours after, having been for about half an hour in the warm bath after it, from which, when immersed, he always found great relief.

Dr. Mosely, in his publication more than twenty years later, recommends the cutting out of the part bit as the only remedy. He says, " this method of treating the bites of mad animals, if on a part where it can be used, will, I am confident, prevent their fatal effects applied *at any time previous* to the first symptoms, that forerun a general affection, which ends in hydrophobia, and admits of *no remedy*."

Dr. Darwin, in his Zoonomia, speaks of hydrophobia as a fatal disease, and considers it to resemble tetanus, or locked-jaw, in its tendency to convulsion from a distant wound. This similarity between the two diseases may suggest, under certain circumstances, to medical practitioners, the utility of bleeding carried to the length of diminishing spasmodic action, in these dangerous, and too frequently fatal, diseases, tetanus and locked-jaw; and, if it is stopped when fainting occurs, no bad consequences follow, as is shown by Dr. Fothergill, and more particularly by Veitch on Ophthalmia, nay, that it is less detrimental than repeated bleedings, even to a less extent.

Dr. Cullen speaks only generally and doubtfully of any preventative or remedy for hydrophobia.

I have now, I believe, stated sufficient to show that the successful treatment of a well-marked case of hydrophobia, is an event of great import to the public and to the medical profession, and that further facts, properly detailed, either to confirm or to show the inefficacy of the practice here recommended, or to point out any other successful method, should be made known by medical practitioners, to rescue this disease from being an opprobrium to the healing art; and attention should be had, on any recurrence of such an accident, to a person who has been cured of a well-marked disease, to ascertain whether the poison of a rabid animal has obeyed the laws of the inoculated animal poisons, that infect the system after a time by absorption, produce a general affection and febrile disease, and thus render any subsequent innoculation or infection innoxious, which, I think, may be probable. If this can be ascertained, much will be gained in the knowledge of this disease, and I have, in consequence, requested to be made acquainted with whatever may befall Benjamin Mason, whose recovery has suggested this idea.

*Fort St. George*, A. BERRY.

